# Plasticity of brain wave network interactions and evolution across physiologic states

**DOI:** 10.3389/fncir.2015.00062

**Published:** 2015-10-26

**Authors:** Kang K. L. Liu, Ronny P. Bartsch, Aijing Lin, Rosario N. Mantegna, Plamen Ch. Ivanov

**Affiliations:** ^1^Laboratory for Network Physiology, Department of Physics, Boston UniversityBoston, MA, USA; ^2^Department of Neurology, Beth Israel Deaconess Medical Center, Harvard Medical SchoolBoston, MA, USA; ^3^Department of Physics, Bar-Ilan UniversityRamat Gan, Israel; ^4^Department of Mathematics, School of Science, Beijing Jiaotong UniversityBeijing, China; ^5^Dipartimento di Fisica e Chimica, Viale delle Scienze, University of PalermoPalermo, Italy; ^6^Center for Network Science and Department of Economics, Central European UniversityBudapest, Hungary; ^7^Division of Sleep Medicine, Brigham and Women's Hospital, Harvard Medical SchoolBoston, MA, USA; ^8^Institute of Solid State Physics, Bulgarian Academy of SciencesSofia, Bulgaria

**Keywords:** neural plasticity, network physiology, brain wave interactions, time delay stability, sleep

## Abstract

Neural plasticity transcends a range of spatio-temporal scales and serves as the basis of various brain activities and physiologic functions. At the microscopic level, it enables the emergence of brain waves with complex temporal dynamics. At the macroscopic level, presence and dominance of specific brain waves is associated with important brain functions. The role of neural plasticity at different levels in generating distinct brain rhythms and how brain rhythms communicate with each other across brain areas to generate physiologic states and functions remains not understood. Here we perform an empirical exploration of neural plasticity at the level of brain wave network interactions representing dynamical communications within and between different brain areas in the frequency domain. We introduce the concept of time delay stability (TDS) to quantify coordinated bursts in the activity of brain waves, and we employ a system-wide Network Physiology integrative approach to probe the network of coordinated brain wave activations and its evolution across physiologic states. We find an association between network structure and physiologic states. We uncover a hierarchical reorganization in the brain wave networks in response to changes in physiologic state, indicating new aspects of neural plasticity at the integrated level. Globally, we find that the entire brain network undergoes a pronounced transition from low connectivity in Deep Sleep and REM to high connectivity in Light Sleep and Wake. In contrast, we find that locally, different brain areas exhibit different network dynamics of brain wave interactions to achieve differentiation in function during different sleep stages. Moreover, our analyses indicate that plasticity also emerges in frequency-specific networks, which represent interactions across brain locations mediated through a specific frequency band. Comparing frequency-specific networks within the same physiologic state we find very different degree of network connectivity and link strength, while at the same time each frequency-specific network is characterized by a different signature pattern of sleep-stage stratification, reflecting a remarkable flexibility in response to change in physiologic state. These new aspects of neural plasticity demonstrate that in addition to dominant brain waves, the network of brain wave interactions is a previously unrecognized hallmark of physiologic state and function.

## 1. Introduction

Physiological systems and organisms exhibit complex dynamics that continuously change with transitions across physiologic states and conditions by adapting neuronal regulatory mechanisms to optimize function and adequately respond to internal and external stimuli (Ivanov et al., 1999; Kantelhardt et al., 2002; Karasik et al., 2002; Ivanov et al., 2007; Schmitt et al., 2009; Schumann et al., 2010; Bartsch et al., 2012). The ability to perform variety of functions and to adapt to short-term and long-term changes in the environment results from high degree of plasticity in the nervous system. Neural plasticity occurs at multiple levels of organization and over a broad range of time scales (Destexhe and Marder, 2004). At the microscopic level, the ability of neurons to react to different inputs and in response to generate various output dynamics serves as a fundamental building block for a broad range of brain functions (Tononi and Cirelli, 2014). Neurons communicate with each other through chemical and electrical synapses, and the temporal firing patterns of presynaptic neurons, various neuropeptides, and neuromodulator neurons (Marder and Thirumalai, 2002) as well as the specific timing of activation of presynaptic and post-synaptic neuron activity modulate synaptic strength (Abbott and Nelson, 2000; Sjöström and Nelson, 2002) and induce synaptic plasticity that further empowers functional variation. Changes in the intrinsic firing properties of individual neurons (Marder et al., 1996) as well as modulation of synaptic strength (Kandel, 2001) play important role in the mechanisms underlying neuronal circuit plasticity (Abeles, 1991; Katz and Frost, 1996; Engel et al., 2001), which in turn affects the integrative properties of neurons leading to transitions in large network behavior. Thus, as a direct consequence of plasticity at the neuronal, synaptic, and circuitry level a variety of brain waves emerge with different dominant rhythms (Steriade et al., 1993; Traub et al., 2001; Steriade and Timofeev, 2003; Hill and Tononi, 2005; Millman et al., 2010; Olcese et al., 2010).

At the integrated system level, plasticity and neuromodulation have crucial roles in altering excitability in the brain and regulating physiological states such as sleep and wake (Gorgoni et al., 2013). Such modulation of excitatory and inhibitory brain activity during different physiologic states and conditions leads to specific brain waves with dominant frequencies—e.g., higher frequency α-wave associated with higher excitability and dis-synchronous cortical activation during quiet wake, and low frequency δ-wave associated with low excitability and global synchronous activation during deep sleep (Kryger et al., 1994; Massimini et al., 2005; Niedermeyer and da Silva, 2005; Siegel, 2005). In addition to dominant brain waves, physiological states are characterized by specific signatures in the temporal modulation of brain waves (Linkenkaer-Hansen et al., 2001; Poil et al., 2008) and their synchronization across different locations (White et al., 1998; Kopell et al., 2000).

The majority of studies in the field have focused on mechanisms of neural plasticity at the microscopic level of neurons, synapses (Jahnke et al., 2008; Deger et al., 2012; Helias et al., 2014) and neuronal circuits (Vardi et al., 2013, 2015), and on the role particular brain waves play in modulating neural plasticity (Diekelmann and Born, 2010). A fundamental question is how brain waves, which carry important information on physiologic function and result from complex mechanisms of neural plasticity, dynamically communicate with each other and collectively coordinate as a network to produce physiologic states. Here we investigate how neural plasticity is manifested through dynamic interactions of brain waves across different frequency bands and brain locations, and how these interactions facilitate physiologic functions. Since neural plasticity at the microscopic level can be modulated by physiologic states (Stefan et al., 2000; Wolters et al., 2005; Schmidt et al., 2006; Huber et al., 2007), thus leading to changes in physiologic function, we hypothesize that transitions across physiologic states would not only lead to changes in dominant brain waves and their temporal organization but will also modulate the coupling between different brain waves and the entire network of their interactions. Further, we hypothesize that the network dynamics of brain wave interactions and their evolution across physiologic states will provide (i) new information on functional plasticity and (ii) a new hallmark of physiological state derived from the network of brain wave interactions.

To address the fundamental question of how different brain waves dynamically communicate as a network, we develop a novel approach inspired by the new interdisciplinary field of Network Physiology (Bashan et al., 2012; Bartsch and Ivanov, 2014; Ivanov and Bartsch, 2014). This field focuses on understanding complex physiologic function as an emergent phenomenon from network interactions among diverse dynamical systems (network nodes). To identify and quantify networks of brain wave interactions, we introduce the concept of time delay stability (TDS) to analyze coordinated bursts in brain wave activity within different physiologically-relevant frequency bands and across different brain areas. Specifically, we investigate how networks of brain wave interactions evolve with different sleep stages (deep sleep, light sleep, rapid eye movement sleep, and wake) because: (i) sleep stages are well-defined physiologic states (Kryger et al., 1994) with external environmental inputs significantly reduced; (ii) sleep is essential for neural plasticity at the microscopic level (Huber et al., 2008); and (iii) disrupting sleep influences motor behaviors, vigilance, and cognitive functions at the integrated system level (Stickgold, 2005; Born et al., 2006; Rye et al., 2012).

Moreover, recent sleep investigations indicate a previously unknown non-homeostatic, non-equilibrium process that underlies the micro-architecture of sleep-stage and arousal transitions on time scales from seconds to hours, where inhibitory and excitatory brain activations appear to be coupled through a common self-organized criticality (SOC) type mechanism (Lo et al., 2002, 2004, 2013). Based on these observations we hypothesize that as a result of the underlying neuronal plasticity, brain waves coordinate their bursting activities across different frequency bands and brain locations to generate distinct physiologic functions. We further hypothesize that networks representing diverse brain wave interactions carry important information about the nature of physiologic function during specific physiologic states, and that the evolution of such brain wave networks across physiologic states reveals new aspects of functional plasticity.

The aim of this study is to bridge the gap between (i) investigations of neuronal and synaptic plasticity on the microscopic level and on mechanisms of vertical integration to generate specific brain waves in neuronal networks, and (ii) phenomenological studies of physiologic states and functions at the macroscopic system level. The Network Physiology approach we present here fills in this gap through investigations of the horizontal integration among brain waves with different physiologic function, and by probing how physiologic states modulate the network of brain wave interactions across physiologically-relevant frequency bands and brain areas. This study provides first insights on the collective network behavior of diverse brain waves, and how such networks undergo complex reorganization with transitions across distinct physiologic states (such as sleep stages) to facilitate advanced brain functions and physiologic regulation.

## 2. Materials and methods

### 2.1. Statement of ethical approval

The data we used in this work are pre-existing multi-channel physiologic recordings from EU SIESTA databases. The detailed protocol of the SIESTA database can be found in Klösch et al. (2001). All participants provided written informed consent.

The research protocol was approved by the Institutional Review Boards of Boston University (Boston, MA, USA) and was conducted according to the principles expressed in the Declaration of Helsinki.

### 2.2. Subject data

We analyze continuously recorded multi-channel physiological data obtained from 36 healthy young subjects (18 female, 18 male, with ages between 20 and 40, average 29 years) during night-time sleep (average record duration is 7.8 h). We focus on physiological dynamics during sleep as sleep stages are well-defined physiological states, and external influences due to physical activity or sensory inputs are reduced during sleep. Sleep stages are scored in 30 s epochs by sleep lab technicians based on standard criteria (Rechtschaffen and Kales, 1968). Specifically, we analyze EEG data from six scalp locations (frontal left—Fp1, frontal right—Fp2, central left—C3, central right—C4, occipital left—O1, and occipital right—O2). In order to compare these very different signals with each other and to study interrelations between them, we extract the following time series from the raw EEG signals: the spectral power of seven frequency bands of the EEG in moving windows of 2 s with a 1 s overlap: δ (0–4 Hz), θ (4–8 Hz), α (8–12 Hz), σ (12–16 Hz), β (16–20 Hz), γ_1_ (20–34 Hz), and γ_2_ (34–100 Hz).

### 2.3. Time delay stability

One key aspect of neuronal plasticity and synaptic plasticity is the time-dependent behavior of single neurons and their synaptic connections (Buzsáki et al., 2012). Heterogeneous distribution in the response latency and characteristic times scales of various neurons and synapses generate complex temporal dynamics in electrophysiological signals including bursting oscillations and long-range correlated brain waves (Denker et al., 2004).

The complexity in the output EEG signals characterized by non-stationarity, continuous fluctuation poses a great challenge in identifying and quantifying dynamical interactions between EEG signals. This challenge is further compounded by the intrinsic time delay between neuronal activity that emerges at different brain locations, which makes traditional cross-correlation techniques inadequate. Here, we adapt a new framework from Network Physiology to analyze brain network interactions based on the concept of Time Delay Stability (TDS), a method tailor-made for quantifying interactions through the output signals of diverse systems (Bashan et al., 2012; Bartsch et al., 2014).

**Figure 1:**
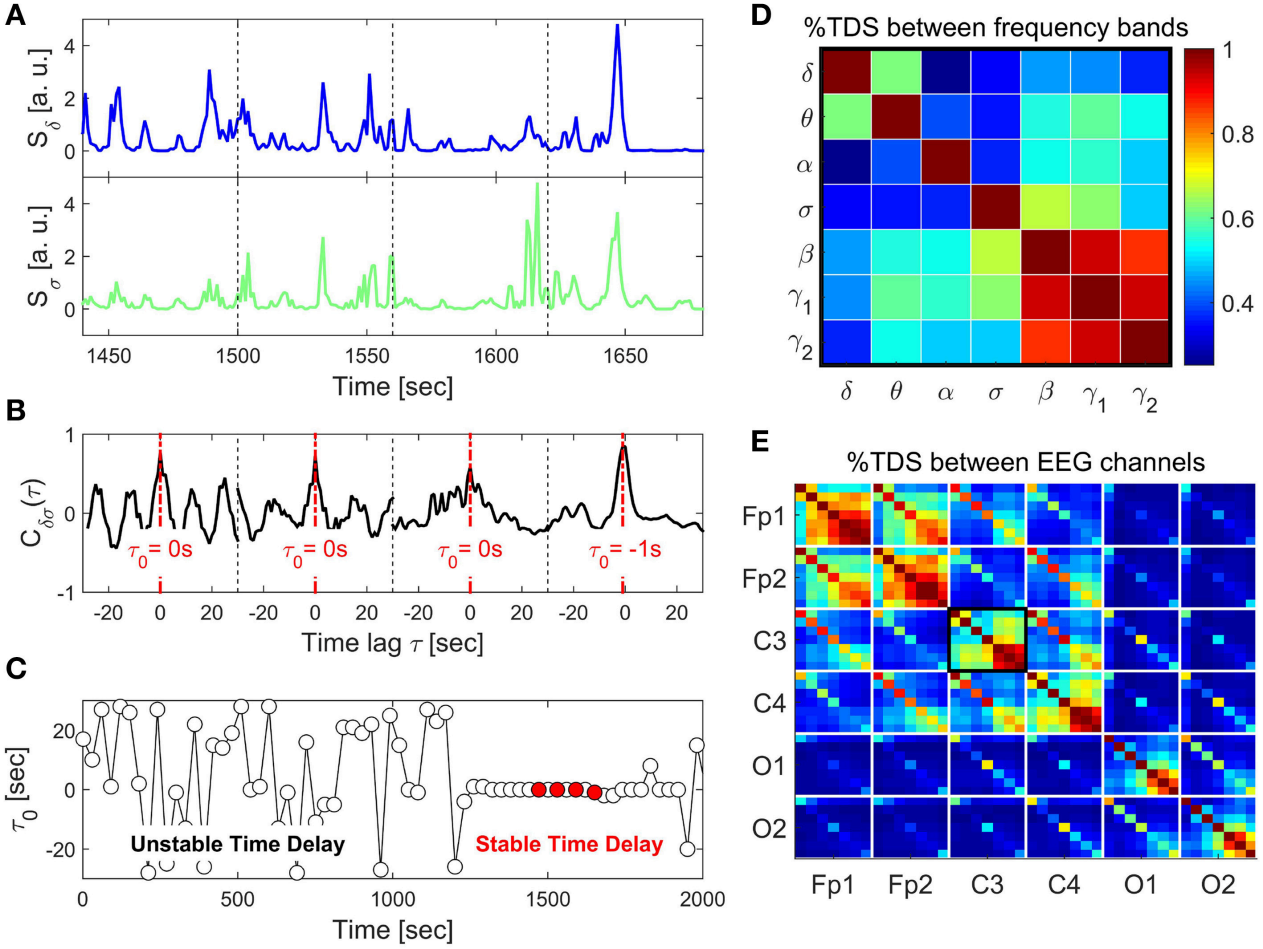
**Schematic presentation of the Time Delay Stability (TDS) method and TDS matrix representing the degree of coupling between different frequency bands across brain locations**. **(A)** Segments of brain EEG power spectra *S*_δ_ and *S*_σ_ for the δ- and σ-band shown for four consecutive 60 s time windows. **(B)** Coordinated bursts in *S*_δ_ and *S*_σ_ lead to pronounced cross-correlation *C*_δσ_ within each time window. The time lag τ_0_ that corresponds to the peak in the cross-correlation function *C*_δσ_(τ) represents the time delay between the two signals. **(C)** Time delay τ_0_ between *S*_δ_ and *S*_σ_ plotted as a function of time for consecutive 60 s windows moving with a step of 30 s. Four red dots represent τ_0_ for the four windows shown in the above panels. Note the transition at ~1200 s from a segment with strongly fluctuating τ_0_ to a stable time delay regime with τ_0_ ≈ constant. Such regime of time delay stability (TDS) indicates the onset of physiological coupling. The fraction of time when TDS is observed in the EEG recording, i.e., % TDS, quantifies the degree of coupling strength. Longer periods of TDS between *S*_δ_ and *S*_σ_ reflect stronger coupling. **(D)** TDS matrix representing the degree of coupling between different physiologically relevant EEG frequency bands (δ, θ, α, σ, β, γ_1_, γ_2_) derived from the C3 channel. Matrix elements represent % TDS, where the color code is shown in the vertical bar. **(E)** Block-matrix representing the degree of TDS coupling between EEG channels (Fp1, Fp2, C3, C4, O1, O2) and between EEG frequency bands. Each off-diagonal block element corresponds to a specific pair of EEG channels and each diagonal block element represents the coupling between different frequency bands within the same EEG channel, as shown in **(D)**.

To probe the interaction between EEG spectral power in two frequency bands A and B, we consider their output signals {*a*} and {*b*} each of length *N*. We divide both signals {*a*} and {*b*} into *N*_*L*_ overlapping segments ν of equal length *L* = 60 s. We choose an overlap of *L*∕2 = 30 s, which corresponds to the time resolution of conventional sleep-stage-scoring epochs, and thus *N*_*L*_ = [2*N*∕*L*] − 1. Next, we calculate $C_{ab}^{\nu }\left(\tau \right)=\frac{1}{L}\overset{L}{\underset{i=1}{\sum }}a_{i+\left(\nu -1\right)L∕2}^{\nu }b_{i+\left(\nu -1\right)L∕2+\tau }^{\nu }$, which is the cross-correlation function within each segment ν ∈ [1, *N*_*L*_] by applying periodic boundary conditions. For each segment ν, we define the time delay $\tau_{0}^{\nu }$ to correspond to the maximum in the absolute value of the cross-correlation function $C_{ab}^{\nu }\left(\tau \right)$ in this segment (Figure 1).

Time periods of stable interrelation between two signals are represented by segments of approximately constant τ_0_ in the newly defined series of time delays, $\left\{\tau_{0}^{\nu }\right\}{|}_{\nu \in \left[1,N_{L}\right]}$. In contrast, absence of stable coupling between the signals corresponds to large fluctuations in τ_0_. We identify two systems as linked if their corresponding signals exhibit a time delay that does not change by more than ±1 for several consecutive segments ν. We track the values of τ_0_ along the series $\left\{\tau_{0}^{\nu }\right\}$: when for at least four out of five consecutive segments ν (corresponding to a window of 5 × 30 s) the time delay remains in the interval [τ_0_ − 1, τ_0_ + 1], these segments are labeled as stable. This procedure is repeated for a sliding window with a step size one along the entire series $\left\{\tau_{0}^{\nu }\right\}$. The % TDS is finally calculated as the fraction of stable points in the time series $\left\{\tau_{0}^{\nu }\right\}$ (Figure 1). We have tested several different values for the window size *L*, i.e., *L* = 30, 60, 120, and 180 s with non-overlapping windows as well as window overlaps *L*∕2 and *L*∕4. The overall TDS results were not significantly different for the different combinations of *L* and overlap, however, there was a tendency to noisier τ_0_ vs. *t* signals for shorter windows and less overlap. On the other hand larger windows reduce the time resolution of the TDS. We chose *L* = 60 s with an overlap of 30 s because this choice was most sensitive to sleep-stage transitions that are scored in 30 s epochs (Figure 1), consistent with the traditional sleep research time scales.

### 2.4. Visualization approach and TDS brain-networks

To visually inspect and dissect the complex communications between one brain area and others that are mediated through various frequency bands, these interactions are mapped onto a network where group averaged TDS links strength are scaled with the line thickness of the corresponding network links.

Network nodes with different colors represent seven different frequency bands (δ, θ, α, σ, β, γ_1_, γ_2_). Each set of seven nodes ordered as a heptagon forms a vertex on the hexagon representing six EEG channels from particular brain locations: 2 Frontal areas (Fp1 and Fp2), 2 Central areas (C3 and C4), and 2 Occipital areas (O1 and O2).

These networks consists of intra-channel links at a chosen channel/location and inter-channel links between the chosen channel location and other brain areas. The intra-channel links for a given channel location are plotted using a gray color code where the darkness and the width of the line are scaled with the link strength. The inter-channel links are plotted with the color representing the frequency band located at the chosen channel (C3, Fp1, or O1) and the line thickness is scaled with the link strength.

If an inter-channel link ends at a node with the same color as the link itself, this link represents interactions between spectral power of the same frequency bands at different locations. We define these links as same-frequency links. Otherwise, links whose color do not match with the node color represent communications between two different frequency bands, and thus are defined as cross-frequency links.

## 3. Results

### 3.1. Plasticity in brain wave interactions across the entire brain

Utilizing our TDS method and network approach, we identify and quantify coupling between brain waves defined by physiologically-relevant EEG frequency bands. We build a network of brain wave interactions, where network nodes represent diverse brain waves at different brain locations and network links represent the strength of TDS coupling between brain waves across the entire brain.

**Figure 2:**
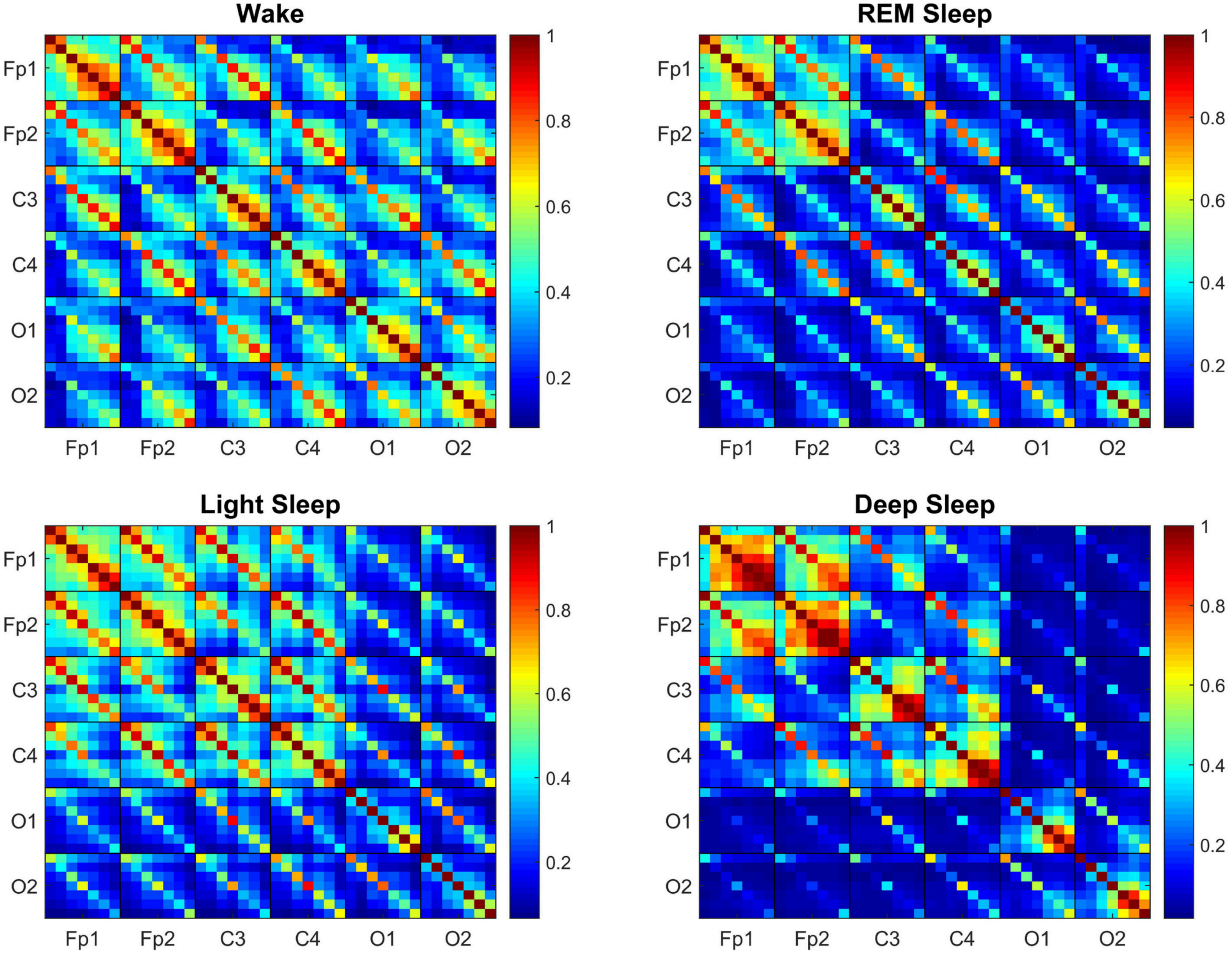
**Transitions in the Time Delay Stability (TDS) matrix across physiologic states indicate neural response and plasticity in brain wave interactions at the integrated level**. Group-averaged Time Delay Stability (TDS) block-matrices during distinct physiologic states (sleep stages) obtained from continuous 8-h EEG recordings during sleep from 36 healthy subjects. Color code represents the average strength of TDS coupling between brain waves (distinct EEG frequency bands) across different brain locations as quantified by % TDS—i.e., the fraction of time out of the total duration of a given sleep stage throughout the night when TDS is observed. Parallel diagonal lines across each off-diagonal matrix block indicate strong coupling between the same EEG frequency band derived from different EEG-channel locations (e.g., coupling of δ_C3_–δ_C4_, α_O1_–α_O2_ etc.). This behavior is consistently observed for all sleep stages indicating that a significant part of the brain-brain interactions across different brain areas are mediated through coupling between the same frequency bands. Transitions in the TDS matrix across different sleep stages are associated with reorganization in brain-network connectivity as reflected by (i) different configurations in the strength of the off-diagonal matrix elements that represent coupling between different EEG frequency bands in both the diagonal and off-diagonal matrix blocks, and (ii) the overall strength of coupling (% TDS, shown by color code)—lower connectivity during Deep Sleep and REM, higher during Light Sleep and highest brain-network connectivity in Wake. Such complex reorganization in the strength of brain interactions (represented by network links in the following figures) across different frequency bands and brain locations demonstrates remarkable plasticity of the brain-network in response to change of physiologic state and function.

We find that brain-wave network links exhibit complex patterns in the coupling strength between frequency bands across different brain locations as represented by the TDS block-matrix elements (Figure 2). Moreover, we find that during distinct physiologic states (sleep stages) the entire brain adjusts the configuration and strength of network connections between brain waves leading to a hierarchical network re-organization with transitions across physiologic states. This demonstrates a remarkable neural plasticity in the way brain waves coordinate their bursting activity at the integrated system level to produce physiologic functions associated with each physiologic state. Specifically, the network of brain wave interactions undergoes a pronounced transition from less connected state during Deep Sleep and REM to a highly connected state in Light Sleep and Wake (Figure 2).

Generally, the strongest links in the network structure of brain wave interactions are those links between brain waves of different frequencies at the same EEG-channel location (i.e., intra-channel interactions) as represented by the diagonal matrix blocks in the TDS matrix. In contrast, interactions among brain waves from different EEG-channel locations (i.e., inter-channel links) are weaker as represented by the off-diagonal matrix blocks (Figure 2). Further, considering all inter-channel links that represent interactions between the same brain waves exhibit strongest coupling, as shown by the dominant diagonal elements in each off-diagonal matrix block (Figure 2). These network features are consistently observed for all sleep stages, indicating a universal and robust structure in brain wave interactions independent of physiologic states. Moreover, our results show that a significant part of the brain wave interactions across different brain areas is mediated through the coupling between brain waves in the same frequency bands.

Comparing the TDS matrix for different sleep stages, we find that in each matrix block the most significant change in the network of brain wave interactions occurs for the off-diagonal matrix elements, which represent interactions between brain waves of different frequencies (Figure 2). Such dramatic re-organization in network connectivity and link strength between different brain waves (from either the same or from different brain locations) indicates high degree of neural plasticity and modulation of global cooperative behavior of brain wave interactions to accommodate physiologic function during different physiologic states. Notably, the underlying mechanism of such neural plasticity influences predominantly the interactions between pairs of different brain waves (as demonstrated by the large color variation in off-diagonal matrix elements across sleep stages, Figure 2). Our analyses show that neural plasticity also affects the generally stronger links between same brain waves, although to a smaller degree (smaller color variation of diagonal matrix elements across sleep stages, Figure 2).

**Figure 3:**
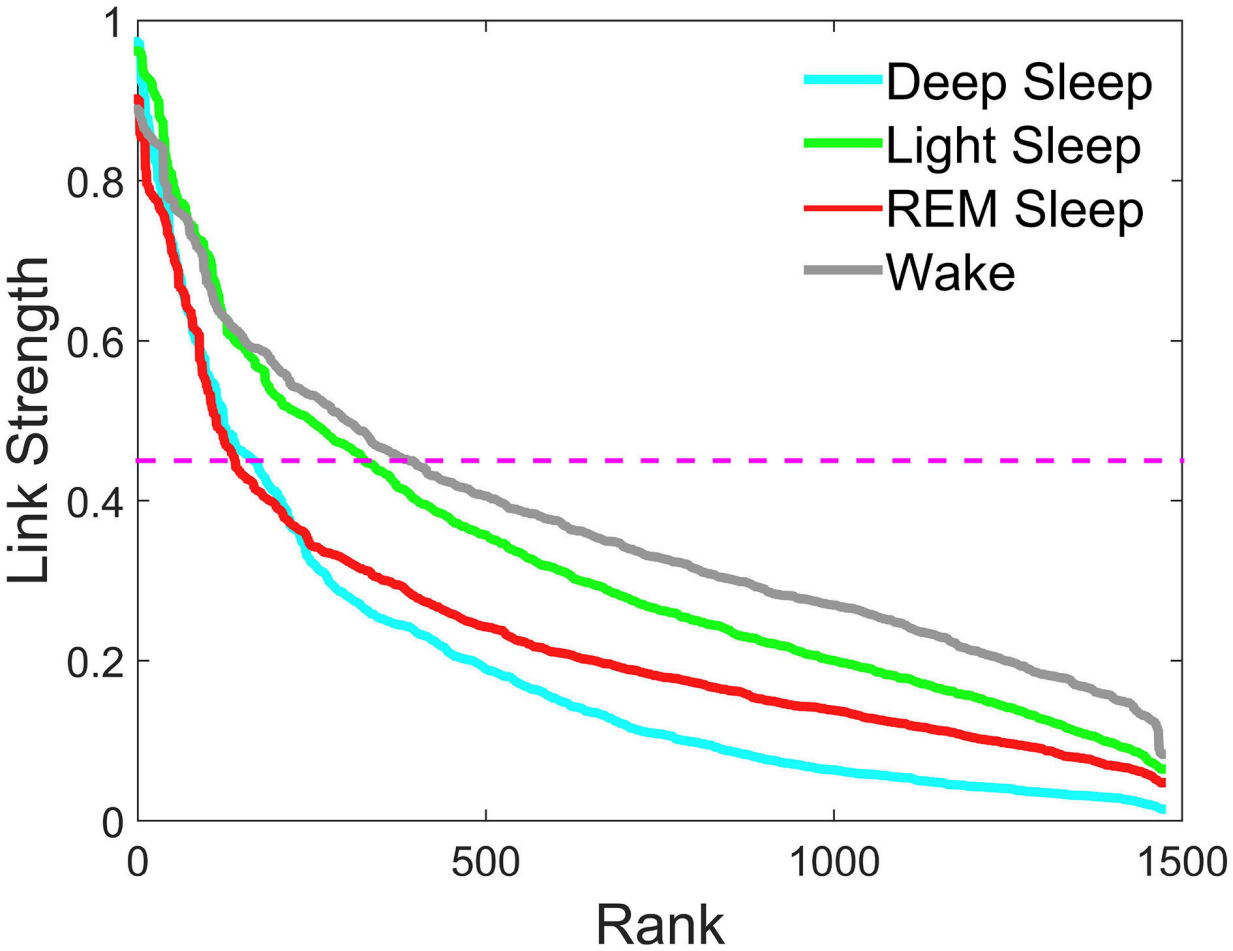
**Rank distribution of the strength of network links connecting different brain waves across brain areas**. Group averaged coupling strength of inter-channel network links between different brain areas represented by the matrix elements in the off-diagonal matrix blocks of the TDS matrix shown in Figure 2 is ranked separately for different sleep stages. Rank 1 corresponds to the strongest link in the network with highest degree of Time Delay Stability (TDS) between a pair of brain waves. The rank distributions for different sleep stages are characterized by different strength of the network links measured in % TDS—consistently lower values for most links during Deep Sleep, higher values during REM and highest during Light Sleep and Wake. Magenta dashed line represents a threshold of 45% TDS. Notably, links above the 45% TDS threshold exhibit two distinct forms of rank distributions—fast decay for Deep Sleep and REM vs. much slower decay for Light Sleep and Wake.

To further assess plasticity in the network of brain wave interactions we compare the rank distribution of the coupling strength of brain-network links connecting brain waves from different EEG-channel locations (inter-channel links). We find that there is a pronounced stratification in the rank distributions with lowest values for most of the brain wave interactions during Deep Sleep, higher during REM and Light Sleep and highest during Wake (Figure 3). Moreover, these rank distributions fall into two separate categories—fast decay for REM and Deep Sleep, and slow decay for Light Sleep and Wake. The separation in two categories is most pronounced for link strength interactions above 45% TDS, where the rank distributions for REM and Deep Sleep as well as the distributions for Light Sleep and Wake practically coincide (Figure 3).

Generally, intra-channel interactions are stronger compared to inter-channel links. In particular, Links associated with Frontal areas have higher strength compared to Central and Occipital areas (Figure 2). Links that represent interactions between the same frequency band have higher strength in general compared to those interactions between different frequency bands, as evidenced by the color code of the diagonal vs. off-diagonal elements in each matrix block for different sleep stages (Figure 2).

### 3.2. Plasticity in brain wave interactions at specific brain locations—hierarchical reorganization of intra-channel and inter-channel interactions

To better understand the role of individual brain areas in the network of brain wave interactions, we consider subnetworks associated with specific brain locations. Specifically, we consider six subnetworks associated with the six EEG channels in the experimental setup (Section 2), where each subnetwork represents the set of network links between different frequency bands (network nodes) derived from a given EEG frequency band and brain waves at all other brain areas (EEG locations).

Our results show a strong and robust symmetry between the left and right hemisphere in both network topology and link strength configurations. For example, we find that for each physiologic state, the subnetwork associated with the C3 channel is almost identical to the one associated with the C4 channel. Thus, we only present and discuss the results obtained for the channels located in the left hemisphere (C3, Fp1, and O1).

#### 3.2.1. Central channel C3

##### 3.2.1.1. Intra-channel network at C3

**Figure 4:**
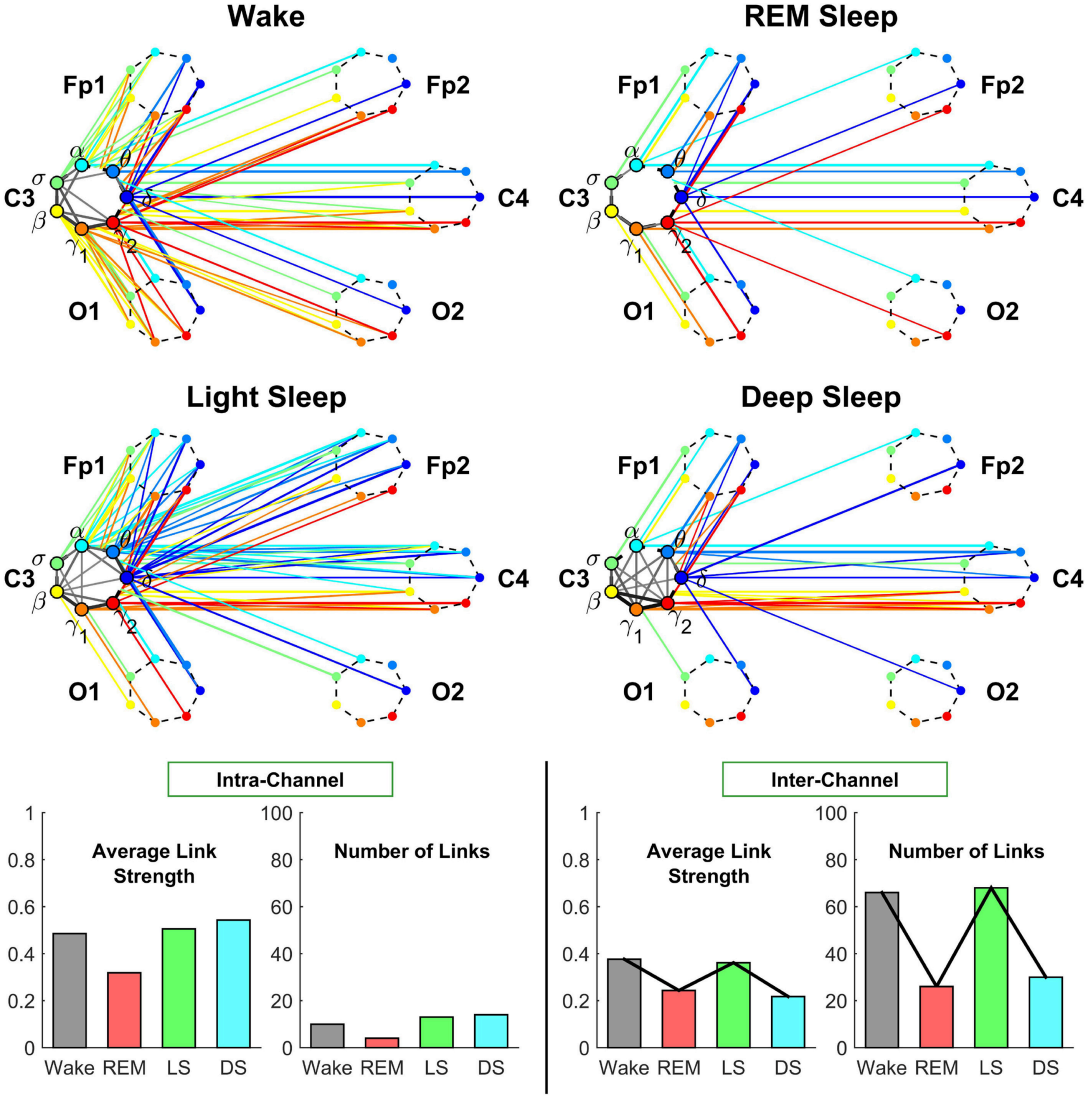
**Neural plasticity represented by transitions in the sub-networks of brain wave interactions centered at the Central C3 channel**. Network nodes with different colors represent seven different frequency bands (δ, θ, α, σ, β, γ_1_, γ_2_) derived from EEG signals. Each set of seven nodes ordered as a heptagon forms a vertex on the hexagon representing six EEG channels from particular brain locations: Two Frontal areas (Fp1 and Fp2), two Central areas (C3 and C4), and two Occipital areas (O1 and O2). Interactions between frequency bands derived from the C3 channel (intra-channel links) are color-coded in gray scale. Interactions between frequency bands derived from the C3 channel and network nodes in all other EEG channels are represented by inter-channel links shown with the same color as the corresponding frequency band (network node) at C3. Line thickness represents the group-averaged link strength as measured by % TDS, and only links with % TDS ≥ 45% are shown (threshold in Figure 3). Both the intra-channel networks (involving links between the frequency nodes at C3) and inter-channel networks (colored links between nodes at C3 and nodes at all other channels) undergo complex hierarchical reorganization across sleep stages, indicating pronounced plasticity in the way frequency bands communicate locally within the C3 Central area and with frequency bands at other brain areas. The intra-channel subnetwork at C3 exhibits low connectivity during REM, medium connectivity during Wake and Light Sleep, and becomes highly connected during Deep Sleep. A similar sleep-stage pattern is also observed for the intra-channel links strength. In contrast, the inter-channel subnetwork between C3 and other brain areas undergoes a very different transition in network connectivity and link strength—from low connectivity in REM and Deep Sleep to high connectivity in Light Sleep and Wake. Note that an identical network structure and reorganization across sleep stages is observed for the Central C4 channel (not shown), indicating a robust symmetry between the left and right hemisphere.

The intra-channel network represents flow of communication (as measured by % TDS, Section 2.3) carried by the spectral power of different frequency bands of the EEG signal recorded at one specific channel location. We find that the connectivity of the intra-channel network at C3 channel changes significantly across distinct physiologic states—we observe a lowest connectivity in REM, a higher one in Wake and Light Sleep and the highest connectivity during Deep Sleep. Notably, in Deep Sleep we observe that most links are associated with high frequency β, γ_1_, γ_2_ bands, which form a dark triangle in Figure 4.

##### 3.2.1.2. Inter-channel network associated with C3

The inter-channel network represents interactions between one channel location and all other brain areas. By investigating the inter-channel network associated with C3, we find that the network undergoes a very pronounced reorganization with transition across sleep stages. This network reorganization, both in network structure and link strength, is more pronounced compared to the reorganization of we observe in the intra-channel network of C3 (Figure 4).

*Central-Central interaction*. Our analyses show that inter-channel links that connect same frequency bands (i.e., same-frequency links) at different central channel locations remain strong (> 45% TDS) during all sleep stages. In contrast, inter-channel links that connect different frequency bands (i.e., cross-frequency links) at the two Central channels C3 and C4 change significantly across different sleep stages. Central-Central cross-frequency links are weak with < 45% TDS during REM (not visible on the network graph), stronger during Deep Sleep and Wake, and reach their maximum strength during Light Sleep as shown in Figure 4.

*Central-Frontal interaction*. We find that inter-channel links representing connections observed between brain waves at the Central and Frontal channels have lower connectivity in REM and Deep Sleep, higher connectivity in Wake and become highly connected during Light Sleep. In general, we find that inter-channel links within the same hemisphere (C3-Fp1) are stronger than cross-hemisphere links (C3-Fp2) in all sleep stages.

*Central-Occipital interaction*. Further we observe that Central-Occipital brain wave interactions are much weaker (lower network connectivity) compared to Central-Central or Central-Frontal brain wave interactions, a behavior which is consistent for all sleep stages (Figure 4). Nonetheless, the connectivity of Central-Occipital subnetwork follows a similar sleep-stage stratification pattern as all other inter-channel subnetworks associated with C3—lower connectivity during Deep Sleep and REM, and higher connectivity during Light Sleep and Wake (Figure 4).

#### 3.2.2. Frontal channel Fp1

**Figure 5:**
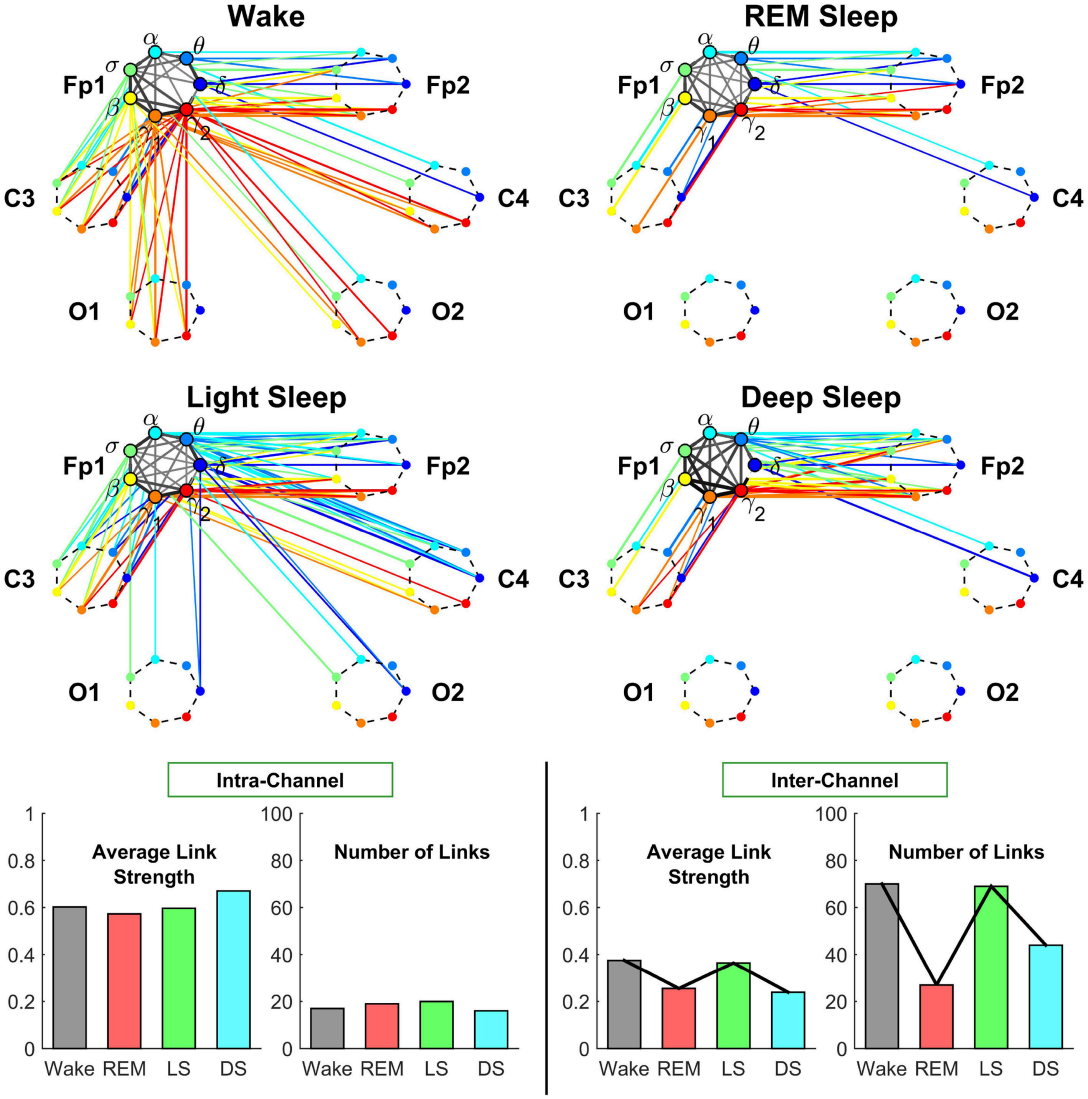
**Neural plasticity represented by transitions in the sub-networks of brain interactions centered at the Frontal Fp1 channel**. Network nodes and network links are illustrated in the same way as described in Figure 4. Line thickness represents the group-averaged link strength as measured by % TDS, and only links with % TDS ≥ 45% are shown. Intra-channel sub-networks (links in gray scale) between different frequency nodes at the Fp1 channel exhibit high connectivity and link strength in all sleep stages. This is in contrast to the pronounced stratification in connectivity and average link strength observed for the C3 intra-channel sub-networks shown in Figure 4, and indicates a reduced plasticity in the intra-channel communications within the Frontal Fp1 area. A very different behavior is exhibited by the inter-channel sub-network representing interactions between frequency nodes at the Fp1 location and frequency nodes at other brain areas, which is characterized by a pronounced reorganization in both global connectivity and link strength configurations: Deep Sleep and REM are characterized by low global connectivity of the inter-channel sub-network and dominant links between Fp1 and the neighboring Frontal and Central areas; in contrast, global connectivity is high during Wake and Light Sleep with emerging Frontal-Occipital links that are predominantly mediated through the high frequency bands during Wake (red color links) and mainly through the low frequency bands during Light Sleep (blue color links). Note that an identical network structure and reorganization across sleep stages is observed for the Frontal Fp2 channel (not shown), indicating a robust symmetry between the left and right hemisphere.

Next we consider the intra- and inter-channel subnetworks associated with the Frontal Channel Fp1. We observe that these subnetworks also exhibit pronounced plasticity, however, undergo a very different reorganization with sleep stage transitions as shown in Figure 5.

##### 3.2.2.1. Intra-channel network at Fp1

Our analyses show that in contrast to the intra-channel network observed for the C3 channel (Figure 5), the intra-channel network at Fp1 remains highly connected across all sleep stages. However, the overall connectivity of the intra-channel networks at Fp1 exhibits a specific sleep-stage stratification pattern with network connectivity and average link strength similar for all sleep stages, which is different from the pattern observed at C3 where network connectivity and link strength are lower during REM and Wake and higher during Light and Deep Sleep.

##### 3.2.2.2. Inter-channel network associated with Fp1

We find that inter-channel network of brain wave interactions associated with Fp1 location exhibits a pronounced pattern with lower connectivity during REM and Deep Sleep and much higher connectivity dying Wake and Light Sleep as shown in Figure 5. Notably, this sleep-stage stratification pattern is very similar to the one we find for the C3 location (Figure 5). Further, we find a similar stratification pattern for the average link strength though it is less pronounced compared to network connectivity (Figure 5).

*Frontal-Frontal interaction*. In contrast to the pronounced sleep-stage stratification pattern in link strength of Central-Central interactions (Figure 4), we find that Frontal-Frontal inter-channel interactions remain strong across all sleep stages for both same-frequency and cross-frequency links (Figure 5). This observation indicates that the frontal area exhibit very different plasticity and functional role in response to sleep stage transitions.

*Frontal-Occipital interaction*. In contrast to Frontal-Frontal, and Frontal-Central brain wave interactions, we find that Frontal-Occipital subnetworks (Fp1-O1 and Fp1-O2) are relatively sparse during Wake and Light Sleep and with practically no connections during REM and Deep Sleep (Figure 5).

Further, we find that during Light Sleep, Frontal-Occipital brain wave interactions are mainly mediated through the relatively low frequency δ, α, σ bands, while in contrast, during Wake, these interactions are mediated through the high frequency bands (β, γ_1_, γ_2_).

#### 3.2.3. Occipital channel O1

##### 3.2.3.1. Intra-channel network at O1

**Figure 6:**
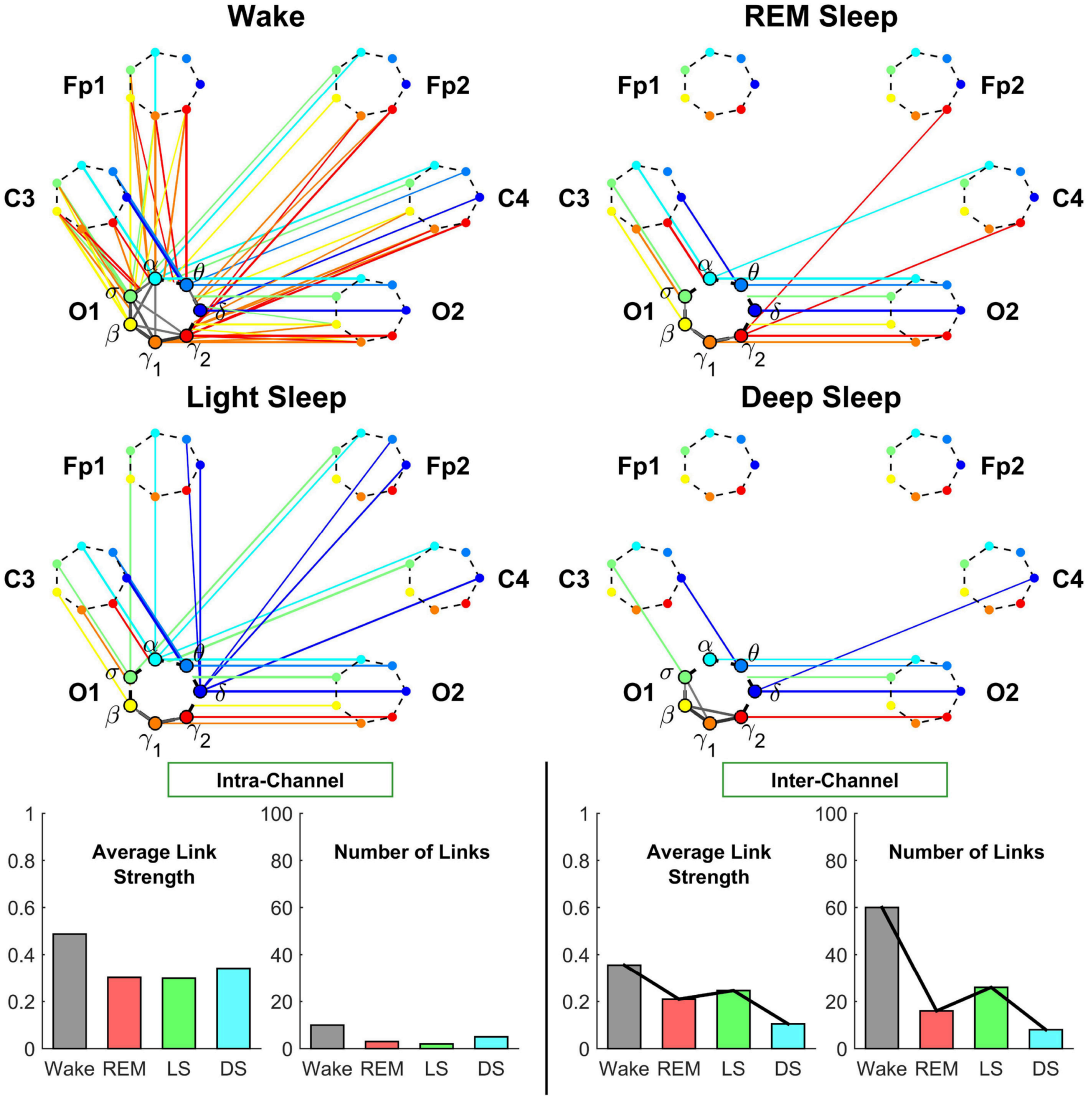
**Neural plasticity represented by transitions in the sub-networks of brain interactions centered at the Occipital O1 channel**. Network nodes and network links are illustrated in the same way as described in Figures 4, 5. Line thickness represents the group-averaged link strength as measured by % TDS, and only links with % TDS ≥ 45% are shown. In contrast to the intra-channel sub-networks of brain interactions across frequency bands within the Central C3 area (Figure 4) and within the Frontal Fp1 area (Figure 5), the intra-channel sub-networks at the Occipital O1 channel have weak links and are generally less connected for all sleep stages—very low connectivity during REM and Light Sleep, slightly higher during Deep Sleep and highest during Wake. In contrast to the intra-channel sub-networks at the Occipital O1 location, the inter-channel sub-networks associated with the O1 channel exhibit pronounced sleep-stage stratification in global connectivity and average link strength—low connectivity during Deep Sleep and REM, and higher connectivity during Light Sleep and Wake, where brain interactions are predominantly mediated through low frequency bands during Light Sleep (blue color links) and through the high frequency bands during Wake (red color links). A similar sleep-stage stratification pattern in the overall connectivity and average link strength of the inter-channel sub-network is consistently observed for the Central C3 and Frontal Fp1 channels (Figures 4, 5) indicating a general rule of network reorganization underlying neural plasticity in brain interactions between frequency bands across brain areas. Note that an identical network structure and reorganization across sleep stages is observed for both O1 and O2 channels, indicating a robust symmetry between the left and right hemisphere that is also observed for the frontal and central channels (Figures 4, 5).

We find that intra-channel network of brain wave interactions at the Occipital O1 location is characterized by low connectivity during Light Sleep and REM, intermediate during Deep Sleep and high connectivity during Wake. We also observe a similar pattern for the average link strength of the O1 intra-channel network (Figure 6).

We note that sleep-stage transitions in the intra-channel network connectivity and average link strength at the O1 location exhibit a very different pattern (Figure 6) compared to the intra-channel networks observed at the C3 and Fp1 location (Figures 4, 5). Moreover, both network connectivity and average link strength at O1 are significantly lower compared to the C3 and Fp1 location.

These observations indicate a remarkable plasticity in brain wave network interactions, where brain wave activations modulate the neural signal at each brain location by varying the interrelation between different frequency bands in response to change in physiologic state. Further, the different level of network connectivity and average links strength, as well as the different patterns of reorganization in brain wave intra-channel interactions we discovered at different brain locations indicate a complex hierarchically structured mechanism of plasticity in brain dynamics to facilitate distinct physiologic functions.

##### 3.2.3.2. Inter-channel network associated with O1

*Occipital-Occipital interaction*. Investigating the inter-channel network of brain wave interactions associated with the O1 brain location, we find that Occipital-Occipital interactions are predominantly mediated through same-frequency inter-channel links in all sleep stages.

In contrast, cross-frequency inter-channel links undergo transitions in links strength across sleep stages—weak links during REM, Deep Sleep and Light Sleep (not shown in Figure 6) and strong links during Wake (above the threshold of 45% TDS, Figure 6) that involve high frequency β, γ_1_, γ_2_ bands. Notably, emergence of strong cross-frequency links between the high frequency bands is consistently observed with transition to Wake for the inter-channel subnetworks associated with Central (Figure 4), Frontal (Figure 5), and Occipital areas (Figure 6).

Occipital-Central and Occipital-Frontal inter-channel brain wave interactions are discussed in the Sections 3.2.1 and 3.2.2 above.

#### 3.2.4. Dominant frequency of inter-channel communications

Our investigations of the subnetworks of inter-channel brain wave interactions associated with the Central (Figure 4), Frontal (Figure 5), and Occipital areas (Figure 6) reveal a consistent pattern, where network connectivity and average link strength are high during Wake and Light Sleep, and lower during REM and Deep Sleep. This stratification pattern represents a previously unknown basic rule of neural plasticity underlying brain wave dynamics. Moreover, this pattern contrasts with the traditional classification of sleep stages where Deep Sleep and Light Sleep are considered similar and are categorized as Non-REM Sleep.

While network connectivity and average link strength of the inter-channel subnetworks show a similarity between Wake and Light Sleep, a closer inspection reveals that during Wake brain wave interactions are predominantly mediated through high frequency β, γ_1_, γ_2_ bands, whereas during Light Sleep most links correspond to interactions between lower frequency δ, θ, α bands.

In summary, our results show that each brain area follows its own rule with respect to the intra-channel communications between different frequency bands. In contrast, brain wave interactions across different locations exhibit a robust sleep-stage stratification pattern in network connectivity and average link strength. These observations suggest the possible presence of two distinct mechanisms of plasticity that regulate the network communications across different physiologic states.

### 3.3. Plasticity of individual brain rhythms and complex transitions in frequency-specific networks across physiologic states

Our investigations show that brain wave interactions between different frequency bands at different brain locations exhibit strong sleep-stage specificity—as represented by dramatic change in the off-diagonal elements of the TDS block matrices (Figure 2) and by cross-frequency links in the Central, Frontal, and Occipital subnetwork during different sleep stages (Figures 4–6). In contrast, brain wave interaction mediated through the same frequency band (e.g., parallel diagonal lines in the TDS block matrices Figure 2) are stronger in all sleep stages compared to the cross-frequency links, indicating an important role of same-frequency links in facilitating communications between different brain locations.

**Figure 7:**
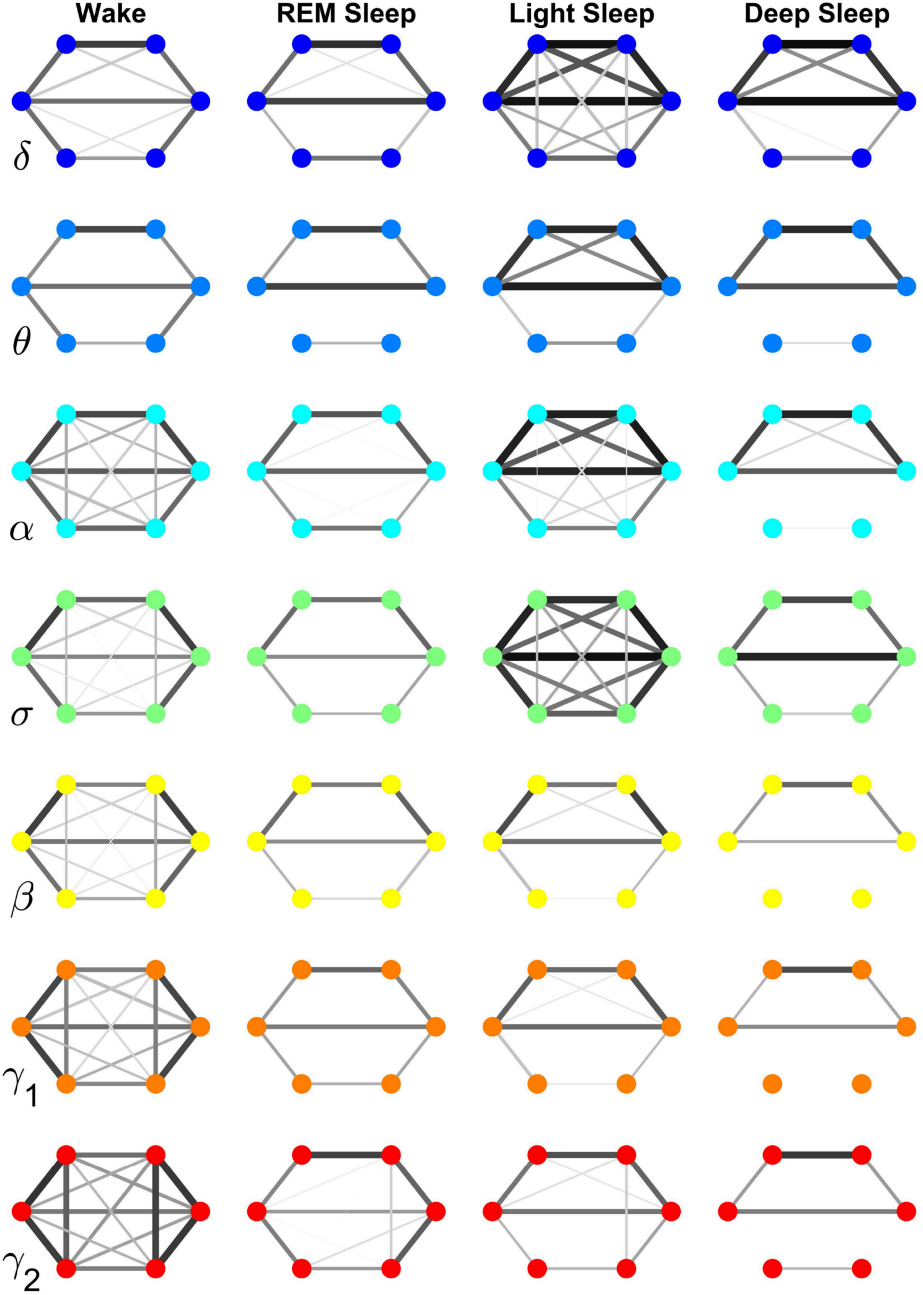
**Neural plasticity in the frequency domain represented by reorganization of network interactions across brain areas mediated through a specific frequency band across physiologic states**. Network nodes represent six different brain areas: Frontal Fp1 and Fp2 (top vertices of the hexagon), Central C3 and C4 (middle vertices), and Occipital O1 and O2 (bottom vertices). Node colors indicate different frequency bands through which the inter-channel brain interactions are mediated. Group-averaged TDS links strength is represented by line thickness and by different color on gray scale. Only links with % TDS ≥ 45% are shown. Brain interactions mediated through specific frequency bands are associated with very different network structure within a given physiologic state, and exhibit distinct patterns of hierarchical reorganization with transition across physiologic states. Specifically, networks representing brain interactions in the δ and σ band are highly connected and with stronger links in Light Sleep, whereas networks representing brain interactions in the α, γ_1_, and γ_2_ band are highly connected and with stronger links during Wake. Notably, network characteristics during Deep Sleep and REM are similar for almost all frequency bands.

To better understand the role of same-frequency interactions across brain areas and how these interactions respond to change in physiologic states, we obtain frequency-specific networks (Figure 7) as the ensemble of inter-channel links connecting a specific frequency band at different brain locations (network nodes). We find that brain interactions mediated through specific frequency bands are (i) associated with very different network structure within a given physiologic state, and (ii) exhibit distinct patterns of hierarchical reorganization with transition across physiologic states (Figure 7).

**Figure 8:**
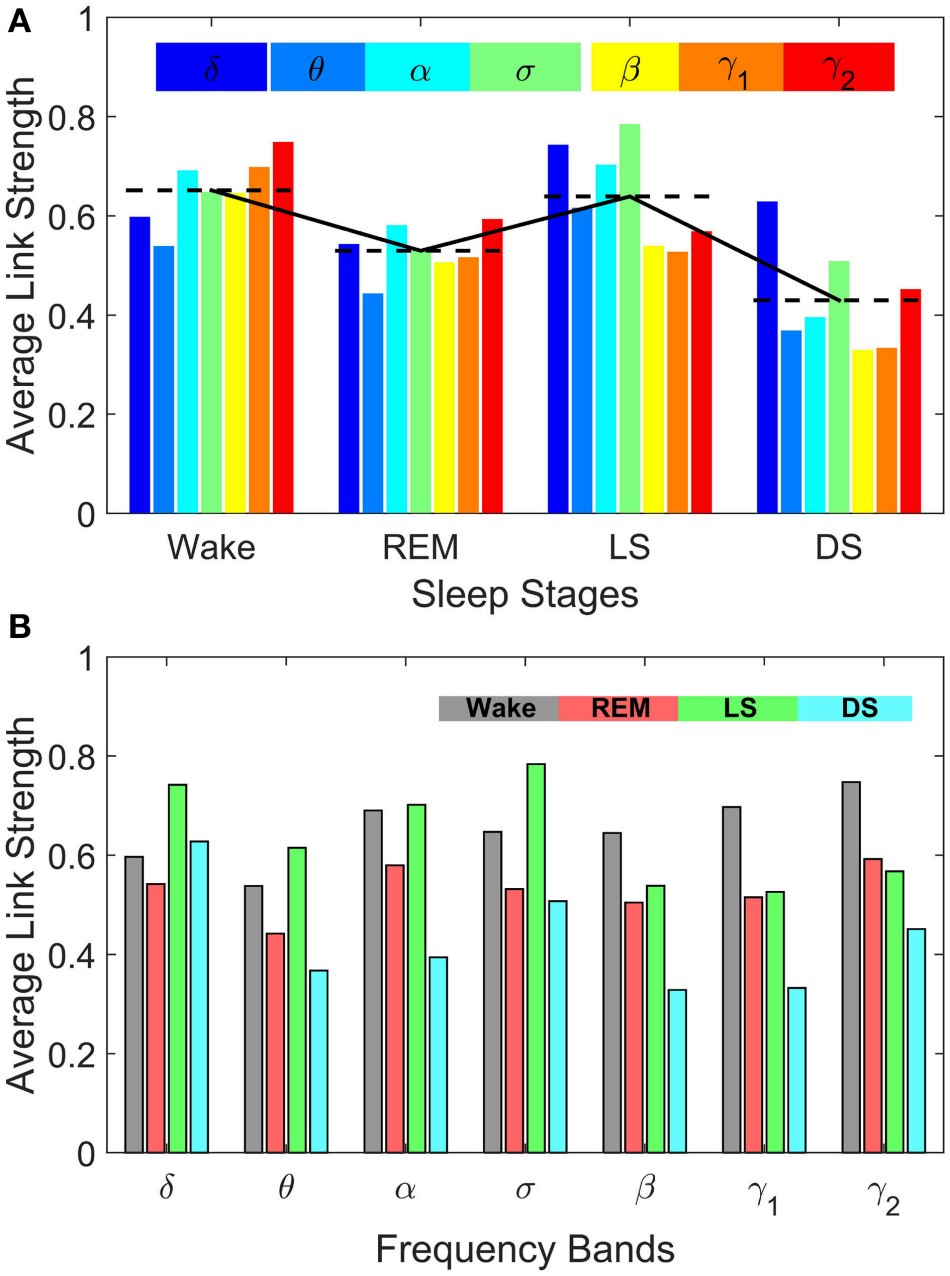
**Sleep-stage stratification patterns in the average strength of inter-channel brain interactions mediated through specific frequency bands**. Average link strength in the frequency-specific networks of inter-channel interactions shown in Figure 7 are grouped by sleep stages **(A)** and by frequency bands **(B)**, respectively. **(A)** demonstrates the degree of involvement of different frequency bands in inter-channel brain communications in each physiologic state (sleep stage). In Deep Sleep inter-channel interactions are mainly mediated through the lowest frequency δ band, while in Light Sleep both the δ band and the mid-frequency σ band associated with sleep spindle activity dominate the interactions between different brain areas. For REM sleep, the average link strength of inter-channel brain networks is similar for all frequency bands. In contrast, during Wake the α band and the high-frequency γ_1_ and γ_2_ bands dominate the inter-channel brain interactions. **(B)** Average link strength of brain network interactions mediated through different frequency bands exhibit distinct sleep-stage stratification patterns. For brain network interactions in the high-frequency β, γ_1_, γ_2_ bands, the average link strength is high during Wake, lower during REM and Light Sleep, and lowest during Deep Sleep. In contrast, the interactions in the mid- and low-frequency bands are characterized by different sleep-stage stratification in the average link strength—indicating pronounced plasticity of brain network interactions in the frequency domain.

Specifically, the network of brain wave interactions across different locations mediated through the δ band is highly connected in Light and Deep Sleep, indicating a strong coordination of bursting activity in the δ band across different brain locations. This is consistent with early reports of global synchronous slow wave brain activity during Non-REM Sleep (Gorgoni et al., 2013). In contrast, the network of the σ band is highly connected during Light Sleep, indicating a strong coordination of spindles at different brain locations. Networks for high frequency β, γ_1_, γ_2_ bands show similar sleep-stage stratification—i.e., highly connected in Wake, and much less connected in REM, Light and Deep Sleep. Notably, there is a general pattern in network connectivity and links strength where networks of all frequency bands are weakly connected during Deep Sleep and REM, and strongly connected during Light Sleep and Wake (Figures 7, 8A). This appears to be a robust sleep-stage stratification pattern, as we also observe it for the inter-channel subnetworks associated with the Central, Frontal and Occipital locations that involve interactions between all frequency bands (Figures 4–6).

Comparing frequency-specific networks within the same physiologic state, we find very different degree of network connectivity and link strength for the different bands. During Deep and Light Sleep, the inter-channel interactions are mainly mediated through the δ and σ band. However, during Deep Sleep the network of δ band is dominant where as during Light Sleep the network of σ band is dominant (Figure 8A). Further, during REM we find that networks of all frequency bands have comparable contributions to the inter-channel brain wave communications, with slight prevalence of network interactions in the α and γ bands (Figure 8A). In contrast, the high-frequency γ_1_ and γ_2_ bands dominates the inter-channel interactions during Wake.

Our investigation reveals that each frequency-specific network is characterized by a different signature pattern of sleep-stage stratification in average link strength, reflecting different neural plasticity of brain activation in different frequency bands. Specifically, we uncover two distinct categories of sleep-stage stratification. For high frequency networks corresponding to β, γ_1_, γ_2_ bands, the average link strength is high during Wake, intermediate (with similar values) during REM and Light Sleep and low during Deep Sleep (Figure 8B). In contrast, for the low and intermediate frequency δ, θ, α, σ bands, the frequency-specific networks are characterized by a different pattern of sleep-stage stratification—higher connectivity and stronger links during Wake and Light Sleep and lower connectivity during REM and Deep Sleep (Figure 8B).

## 4. Summary and discussion

Recent developments in the new field of Network Physiology indicate that complex physiologic functions could be understood through investigating networked communications between diverse dynamical components (Bashan et al., 2012; Bartsch and Ivanov, 2014; Ivanov and Bartsch, 2014). Adapting this integrative approach to multi-channel EEG signals, our investigation reveals a wealth of information about the dynamical interactions between various brain rhythms at different brain locations.

We find that complex network communications between brain waves that encompass not only the coordinated activation across different brain locations within the same frequency band but also previously unexplored interactions across different frequency bands that have been traditionally associated with distinct physiologic states and functions. Our results demonstrate that both network connectivity and strength of brain wave interactions significantly change with transition from one physiologic state to another. This finding indicates that in addition to the traditional view defining physiologic state and function through the prism of a distinct or dominant brain rhythm, networks of interactions across different brain waves and locations are a fundamental signature of physiologic states.

Our observations that brain wave network interactions evolve with transition from one physiologic state to another following a particular pattern of reorganization indicate a new aspect of neural plasticity reflected in the coordinated activation of different brain rhythms across brain areas. Specifically, the plasticity of brain wave network interaction is manifested at three levels of brain dynamics:

1. *Globally:* the entire network of brain interactions across all brain locations and frequency bands undergoes a pronounced transition from a less connected state during Deep Sleep and REM to a highly connected state in Light Sleep and Wake.
2. *Locally:* each brain area has its own characteristics pattern of network interactions between brain waves for a given physiologic state. Further, network interactions associated with a particular brain location exhibit specific pattern of reorganization with transitions across physiologic states.
3. *In the frequency domain:* networks of interactions across brain areas that are mediated through the same frequency band are characterized by different structure and link strength, and exhibit different reorganization patterns across sleep stages.

These observations indicate a complex and hierarchical organization in brain wave interactions that changes in response to different physiologic states. Specifically, we find that intra-channel networks representing interactions between different brain rhythms at the same location exhibit location-specific patterns of reorganization across physiologic states. In contrast, the inter-channel networks exhibit a universal pattern of sleep-stage stratification for the Central, Frontal and Occipital areas that is characterized by high connectivity and link strength during Wake and Light Sleep, and low connectivity and link strength during REM and Deep Sleep.

Compared to other kinds of dynamical and functional brain maps constructed from fMRI, MEG, or BOLD signals (Bullmore and Sporns, 2009), and recent investigations on the structural-functional relationship at the neural circuits level (Bonifazi et al., 2009) as well as at the integrated brain level (Diez et al., 2015), our approach based on the TDS method focuses on the coordinated bursting activity of brain waves in different frequency domains. Generally, this integrative approach allows us to investigate how multi-component dynamical systems self-organize as a result of network interactions among components in order to generate complex functions, and to elucidate the mechanism underlying the evolution of system dynamics across states and conditions. The presented here approach provides a new kind of detailed brain maps allowing us to track the evolution and dynamics of brain functional states in real time, and can be broadly applied to quantify brain wave network interactions under different neuropathological conditions to derive novel biomarkers based on the brain network characteristics. Indeed, our new observations of similarity in network structure between REM and Deep Sleep, and between Wake and Light Sleep provide new insight to traditional sleep research, where Light Sleep and Deep Sleep are often considered similar and classified together as a Non-REM state. Further, in contrast to traditional interpretation of α rhythms as being irregular, localized, and desynchronized, and associated with arousals and quiet wake (Kryger et al., 1994; Siegel, 2005; Born et al., 2006), our findings demonstrate a robust network representing coordinated bursts of α waves activation across different brain locations, as well as a distinct pattern in the evolution of α wave network interactions with transition across sleep stages.

Our observations trigger new questions about the role of neural plasticity not only in generating specific brain rhythms but also in facilitating brain wave communications globally and locally, as well as across different frequency bands. Since networks of local and global brain wave interactions exhibit distinct characteristics, our findings raise the hypothesis that different mechanisms of neural plasticity may be responsible for the local or global aspect of brain rhythms communications. Our work demonstrates the utility of the Network Physiology approach in understanding physiologic state and function in the context of network communications between different brain waves.

The reported here findings of plasticity in brain wave network interactions will facilitate further efforts to bridge microscopic mechanisms of neuronal plasticity with emergent brain dynamics at the system level. Current investigations focus on understanding the role of neuronal and synaptic plasticity in generating particular brain rhythms associated with a specific physiologic state and function. Our empirical observations may guide further efforts in exploring mechanisms of neuronal and synaptic plasticity that also account for the complex hierarchical organization of network interactions between brain rhythms.

## Author contributions

RB wrote the algorithm. KL, RB, and AL analyzed the data. KL, AL, PI, and RM participated in the development of the network analysis of TDS matrices. KL, RB, AL, and PI prepared the manuscript. PI initiated the investigation and supervised all aspects of the work. All authors discussed the results and commented on the manuscript.

### Conflict of interest statement

The Editor Ido Kanter declares that, despite being affiliated to the same institution as author Ronny P. Bartsch, the review was conducted objectively. The authors declare that the research was conducted in the absence of any commercial or financial relationships that could be construed as a potential conflict of interest.
